# Plasmid and Host Strain Characteristics of *Escherichia coli* Resistant to Extended-Spectrum Cephalosporins in the Norwegian Broiler Production

**DOI:** 10.1371/journal.pone.0154019

**Published:** 2016-04-25

**Authors:** Solveig Sølverød Mo, Jannice Schau Slettemeås, Einar Sverre Berg, Madelaine Norström, Marianne Sunde

**Affiliations:** 1 Department of Diagnostic Services, Norwegian Veterinary Institute, Oslo, Norway; 2 Department of Foodborne Infections, Norwegian Institute of Public Health, Oslo, Norway; 3 Department of Health Surveillance, Norwegian Veterinary Institute, Oslo, Norway; 4 Department of Bacteriology and Immunology, Norwegian Institute of Public Health, Oslo, Norway; Catalan Institute for Water Research (ICRA), SPAIN

## Abstract

*Escherichia coli* resistant to extended-spectrum cephalosporins have been detected in the Norwegian broiler production, despite the fact that antimicrobial agents are rarely used. The genetic mechanism responsible for cephalosporin resistance is mainly attributed to the presence of the *bla*_CMY-2_ gene encoding a plasmid-mediated AmpC-beta-lactamase (pAmpC). The aim of this study was to characterize and compare *bla*_CMY-2_ containing *Escherichia coli* isolated from the intestinal flora of broilers and retail chicken meat (fillets) to identify possible successful clones and/or resistance plasmids widespread in the Norwegian broiler production. Methods used included PCR based phylotyping, conjugation experiments, plasmid replicon typing, pulsed-field gel electrophoresis, multiple locus variable-number tandem-repeats analysis and whole genome sequencing. The nucleotide sequence of an IncK plasmid carrying *bla*_CMY-2_ was determined. Intestinal isolates displayed a higher degree of genetic diversity than meat isolates. A cluster of genetically related isolates belonging to ST38, phylogroup D, carrying *bla*_CMY-2_ containing IncK plasmids was identified. Furthermore, genes encoding plasmid stability systems (*relBE/stbDE* and *pndAC)* were identified on the IncK plasmid. Single nucleotide polymorphism (SNP) analysis of a subset of isolates confirmed a close genetic relationship within the two most prevalent STs. The IncK plasmids within these two STs also shared a high degree of similarity. Cephalosporin-resistant *E*. *coli* with the same genetic characteristics have been identified in the broiler production in other European countries, and the IncK plasmid characterized in this study showed close homology to a plasmid isolated from retail chicken meat in the Netherlands. The results indicate that both clonal expansion and horizontal transfer of *bla*_CMY-2_ containing plasmids contribute to dissemination of cephalosporin resistant *E*. *coli* in the broiler production. The presence of plasmid stability systems may explain why the IncK plasmid containing *bla*_CMY-2_ is maintained and disseminated in the Norwegian broiler production in absence of selection pressure from the use of antimicrobial agents.

## Introduction

In the last decade, *Escherichia coli* resistant to extended-spectrum cephalosporins have emerged globally in both human and veterinary medicine [1–4]. This trend is concerning, as cephalosporins are defined as critically important for treatment of human infections [5]. Cephalosporin resistance in *E*. *coli* is primarily mediated by the spread and acquisition of genes encoding extended-spectrum beta-lactamases (ESBLs) and/or plasmid-mediated AmpC-beta-lactamases (pAmpC) [6]. These genes are usually located on plasmids [7], and thus have a large potential for dissemination in the bacterial population through horizontal spread. Furthermore, clonal spread also plays a significant role in the dissemination of cephalosporin-resistant *E*. *coli* [2], exemplified by the global spread of *E*. *coli* sequence type 131 (ST131) containing the *bla*_CTX-M-15_ gene [8, 9].

In veterinary medicine, poultry have been associated with high rates of cephalosporin-resistant *E*. *coli* [3]. Recently, cephalosporin-resistant *E*. *coli* have also been detected in the Norwegian broiler production pyramid [10–13]. However, the situation in Norway is unique, as the use of antimicrobials is minimal, and therefore selection pressure is virtually absent [10–13]. Only a single broiler flock was treated with antimicrobial agents in 2013 and 2014 [14, 15], and four flocks were treated in 2015 [16]. Also, a previous study has shown that the *bla*_CMY-2_ gene, encoding a pAmpC-beta-lactamase, is responsible for cephalosporin resistance in the vast majority of the resistant isolates [10]. In other countries, a more heterogeneous genetic background for cephalosporin resistance is present, also including genes of the *bla*_CTX-M_, *bla*_SHV_ and *bla*_TEM_ groups [3]. Furthermore, fresh conventional chicken meat available at retail is produced in Norway, and the import of chicken meat is very limited [17]. Thus, consumers may be exposed to *E*. *coli* carrying *bla*_CMY-2_ through handling of raw chicken meat, or by consumption of undercooked chicken meat. The occurrence of cephalosporin resistant *E*. *coli* in other food products seems to be of less importance in Norway [11–13, 18]. Import of breeding animals and hatching eggs has been suggested as the likely source of the pAmpC-producing *E*. *coli* present in the Norwegian broiler production pyramid [10, 19, 20].

It has been hypothesized that animals and food contaminated with ESBL/pAmpC-producing *E*. *coli* may constitute a reservoir for human acquisition of such bacteria. An increasing number of studies highlight the similarities between isolates and resistance elements from animals and/or meat and humans [4, 21–28]. In order to minimize a possible zoonotic transfer of resistant bacteria, it should be an overall goal to keep the occurrence of ESBL/AmpC-producing *E*. *coli* in animals and food at the lowest possible level.

The aim of this study was to characterize and compare pAmpC-producing *E*. *coli* isolated from the intestinal flora of broilers and retail chicken meat (fillets) in order to identify possible successful clones and/or resistance plasmids widespread in the Norwegian broiler production. Furthermore, the nucleotide sequence of a frequently occurring IncK plasmid carrying *bla*_CMY-2_ was determined. This knowledge is important in order to evaluate a possible “spill-over” effect to humans and the environment, and in evaluation of the epidemiology of *bla*_CMY-2_ carrying plasmids in the Norwegian and European broiler production.

## Materials and Methods

### Bacterial isolates

A total of 232 *E*. *coli* with pAmpC-production previously collected within the frame of the Norwegian monitoring programme for antimicrobial resistance in bacteria from food, feed and animals (NORM/NORMVET) were included in the study. The isolates were collected from the intestinal flora of healthy broiler chickens by the use of boot swabs and from retail chicken meat (fillets) in 2011 (n = 108, boot swabs), 2012 (n = 66, retail meat) and 2014 (n = 58, retail meat). Methods for isolation and species determination are described previously [11–13]. The included isolates represented all pAmpC-producing *E*. *coli* isolated from broiler in 2011 and retail chicken meat in 2012 and 2014. All isolates were known to harbour the *bla*_CMY-2_ gene. Intestinal isolates originated from pieces of boot swabs collected in the Norwegian *Salmonella* control programme for live animals. Five samples were collected each week during January to November 2011. One piece of boot swab was analysed per flock, and maximum one isolate was obtained per flock. Meat isolates originated from samples collected at retail in counties Oslo, Akershus and Vestfold following a proportionate stratified sampling scheme throughout 2012 and 2014, representing the market share from the three largest chicken meat producers in Norway. Due to the centralised structure of the Norwegian meat production, samples collected in the three counties represent production units in all parts of Norway with broiler production. The chicken meat producers are affiliated to different supermarkets rather than different parts of the country and a representative proportion of samples from each meat producer according to their market shares were obtained by collecting samples from different supermarkets. Each meat isolate represent one unique sample. All isolates had been subjected to susceptibility testing (VetMIC or Sensititre® TREK) and the minimum inhibitory concentrations (MICs) to several antimicrobial agents were known [11–13].

### Determination of phylogroups

All isolates were subjected to phylotyping using multiplex PCR as described previously [29]. The isolates were classified into phylogroup A, B1, B2 or D. An isolate belonging to the B2 group, (*E*. *coli* 2003500827) [30] producing amplicons with all four primer sets, was included as positive control in each PCR run.

### Conjugation experiments

Transferability of *bla*_CMY-2_ containing resistance plasmids was determined by conjugation experiments. Quinolone susceptible isolates were subjected to conjugation with quinolone-resistant *E*. *coli* DH5α (CCUG 32825) as the recipient. Quinolone-resistant isolates were subjected to conjugation with *E*. *coli* OneShot^TM^ cells with pCR^TM^ II vector encoding kanamycin resistance (Invitrogen™, LifeTechnologies, Thermo Fisher Scientific Inc., Waltham, MA, USA). Overnight cultures of recipient and donor was mated 500 μL: 10 μL in four mL Luria Bertani (LB) broth (Merck, Damstadt, Germany), and incubated at 37°C. From the respective matings, 100 μL broth was plated out on Mueller Hinton agar (Difco, Becton Dickinson and company, Sparks, MD) supplemented with 0,5 mg/L cefotaxime and 20 mg/L nalidixic acid (*E*. *coli* DH5α) or 0,5 mg/L cefotaxime and 50 mg/L kanamycin (*E*. *coli* OneShot^TM^), and incubated at 37°C for 24–48 hours. Sampling of the matings was done using the following strategy: The first samples were plated out after four hours incubation. If no transconjugants were identified, samples were also plated out after 24 hours incubation. Furthermore, a new mating was prepared and samples plated out after two and six hours if no transconjugants were identified in the first conjugation experiment. Presumptive transconjugants were plated on blood agar and lactose-saccharose-bromthymol blue agar to inspect the colony morphology. All donor strains were lactose fermenters, while the recipient strains did not ferment lactose. In addition, the recipient strain grew with small characteristic colonies on blood-agar. The transconjugants were subjected to Real-Time-PCR with a previously published probe [31] to confirm conjugative transfer of *bla*_*C*MY-2_.

### PCR-based replicon typing

PCR-based replicon typing (PBRT) was performed on all transconjugants to determine the incompatibility group of plasmids transferred from the donor isolates. The replicon typing was carried out using the PBRT KIT-PCR- based replicon typing (Diatheva, Fano, Italy), according to the manufacturers’ instruction. Positive and negative controls were included in each PCR reaction.

### Pulsed-Field gel electrophoresis

All isolates were subjected to Pulsed-Field Gel Electrophoresis (PFGE) after digestion with the *Xba*I restriction enzyme (Sigma, St Louis, MO). A protocol recommended by Pulse Net [32] was used with minor alterations as previously described [30]. Lambda Ladder PFG marker (New England Biolabs, Hitchin, UK) was included in each run. The banding patterns were evaluated using UPGMA cluster analysis in BioNumerics version 6.6 (BioNumerics, Applied Maths, Sint-Marten-Latem, Belgium) with tolerance 1% and optimization 1%, and visual inspection. Banding patterns with ≥97% similarity were defined as identical, while banding patterns with ≥80% similarity were defined as belonging to the same PFGE cluster.

### Multiple locus variable-number tandem-repeat analysis

A subset of meat isolates (n = 20) that were non-typeable by PFGE were subjected to multiple locus variable-number tandem-repeat analysis (MLVA) to investigate the genetic relatedness between these isolates. MLVA of 10 loci was conducted as previously described [33]. The PCR products were separated by capillary electrophoresis on a 3130*xl* Genetic Analyzer (Applied Biosystems Life Technologies, Thermo Fisher Scientific Inc., Waltham, MA). Peaks were analysed using Peak Scanner Software (Applied Biosystems) with respect to colour and size. Allele numbers were determined based on the fragment size. If no fragment was detected, the allele number was set to zero. Intestinal isolates that were non-typeable by PFGE were not subjected to further genotyping.

### Whole genome sequencing

In order to do further characterization of isolates within the two largest clusters identified in this study, we selected relevant isolates for whole genome sequencing (WGS). These included five isolates representing the largest PFGE cluster, of which three isolates were from faeces and two isolates from meat. Also, five isolates from meat displaying a banding pattern highly similar to the largest PFGE cluster were included. Finally, five meat isolates with highly similar MLVA profiles, representing the second largest cluster, were subjected to WGS. One isolate from 2011 and one isolate from 2012 were sequenced twice to serve as controls of reproducibility. DNA was isolated manually by the use of the Wizard Genomic Purification Kit (Promega Corporation, Madison, WI) (isolates from 2011 and 2012) or automated by the use of MagNA Pure LS Total Nucleic acid Isolation kit in a MagNA Pure LS instrument (Roche Diagnostics, Mannheim, Germany) (isolates from 2014). Library construction and WGS was performed by BGI (BGI Tech Solutions Co. Ltd., Hong Kong) and by the use of a HiSeq 2500 Illumina platform. Furthermore, the sequence servicing centre provided trimmed, cleaned paired- end read sets and assembled contigs by use of SOAPdenovo software version 2.04 (http://sourceforge.net/projects/soapdenovo2/files/SOAPdenovo2/). The processed sequence data were analysed *in silico* with regard to multi-locus sequence type (ST) [34], serotype, acquired resistance genes, plasmid replicon types and virulence genes by the use of tools available online at www.genomicepidemiology.org from Center for Genomic Epidemiology (CGE), DTU, Denmark. Also, a SNP tree was constructed using CSI Phylogeny 1.1 [35] available from CGE, to investigate the number of SNP differences between the isolates subjected to WGS. An *E*. *coli* isolated from retail meat in 2012 (2012-01-1292) was used as reference. A selection of *E*. *coli* reference genomes available from NCBI was also included. The analysis was run using default settings.

Furthermore, the nucleotide sequence of one frequently occurring IncK plasmid was reconstructed. The plasmid was present in one of the isolates within the largest PFGE cluster (isolate no 2012-01-1292). A transconjugant from the conjugation experiment containing the plasmid (1292DH5α) was subjected to WGS, and the draft sequence of the plasmid was identified in an assembled contig. This was done by aligning each contig to an assembled sequence of *E*. *coli* DH1 (accession no. CP001637.1) in CLC Genomics (CLC Bio, Qiagen, Aarhus, Denmark). One scaffold did not align to the DH1sequence, and was assumed to represent the IncK plasmid sequence. To ensure sufficient quality of the plasmid sequence, Sanger sequencing was performed to cover areas with inadequate quality, regions containing repeat sequences, and each side of the scaffold to determine if it could be closed into a circular sequence. Plasmid DNA was extracted using Qiagen Plasmid Plus Maxi Kit (Qiagen, Venlo, The Netherlands) and used as template directly in the sequencing reactions. Primers for sequencing relevant regions were designed using CLC Genomics. BigDye Terminator v3.1/1.1. cycle sequencing kit (Applied Biosystems) was used to determine the nucleotide sequences. The reactions were subjected to capillary electrophoresis on a 3130*xl* Genetic Analyser (Applied Biosystems), and further analysis was performed in CLC Main Workbench (CLC Bio, Qiagen). Open reading frames (ORFs) on the plasmid sequence were identified using CLC Genomics. Annotations were performed by a combination of automated annotation using RAST v4.0 [36] and manual annotation in CLC Main Workbench. The plasmid was designated pNVI1292.

To investigate if similar IncK plasmids were present in the other isolates subjected to WGS, we used CSI Phylogeny with pNVI1292 as reference [35].

## Results

### Phylogenetic grouping and genetic relationship

The results from the phylotyping experiments showed that phylogroup D dominated among isolates from both broilers (56%) and meat (84%). Seven isolates from broilers (6%) and six isolate from meat (5%) belonged to phylogroup B2. Furthermore, 31 broiler isolates (29%) and two meat isolates (2%) grouped into phylogroup B1, while nine (8%) broiler isolates and 12 (10%) meat isolates belonged to phylogroup A. The distribution of phylogroups is shown in Table 1.

**Table 1:** Distribution of phylogroups, conjugative *bla*_CMY-2_ containing plasmids and replicon types of conjugative plasmids among pAmpC-producing *Escherichia coli* isolated from broiler faeces and retail chicken meat in Norway 2011, 2012 and 2014.

Origin	Replicon- type	A	B1	B2	D	Total
Broiler 2011	IncK	3	6	1	32	42
	IncI1	3	20	4	25	52
	IncK and IncI1	1	0	2	3	6
	NTP	2	5	0	1	8
Total broiler 2011		9	31	7	61	108
Retail chicken meat 2012	IncK	4	0	1	56	61
	IncI1	1	0	0	0	1
	NTP	0	0	0	4	4
Total retail chicken meat 2012		5	0	1	60	66
Retail chicken meat 2014	IncK	6	0	5	28	39
	IncK and IncI1	1	0	0	0	1
	NTP	0	2	0	16	18
Total retail chicken meat 2014		7	2	5	44	58
**Overall total**		21	33	13	165	232

NTP: Non-transferable plasmid

PFGE and subsequent cluster analysis revealed that the intestinal isolates showed a higher diversity than the meat isolates (S1 and S2 Figs). Furthermore, a main cluster including 69 isolates was identified when all isolates were analysed together. This cluster included 20 of 108 intestinal isolates (2011), and 49 of 124 meat isolates (43 of 66 from 2012 and 6 of 58 from 2014). The main cluster is indicated in the dendrogram based on the PFGE banding pattern of all isolates included in the study (S3 Fig). Within the main cluster, 25 isolates displayed identical banding patterns. Of these, 21 isolates originated from meat and four from the intestinal flora. *E*. *coli* with this banding pattern was isolated from meat samples originating from two different manufacturers, and thus different abattoirs. A proportion of the isolates were non-typeable (n = 50), including 30 intestinal isolates and 20 meat isolates.

Of the 20 meat isolates subjected to MLVA (all non-typeable by PFGE), 15 had highly similar MLVA profiles indicating close genetic relationship. All these isolates belonged to phylogroup D. Of the remaining five isolates, two belonged to phylogroup D, and both displayed unique MLVA profiles. The remaining three isolates grouped into phylogroup A and displayed closely related MLVA profiles, but differed substantially from the other isolates subjected to MLVA (S4 Fig).

### Transferability and replicons of *bla*_CMY-2_ containing plasmids

Conjugative plasmids carrying *bla*_CMY-2_ were identified in 202 of 232 isolates (87%). A similar occurrence of conjugative IncI1 and IncK plasmids was observed among intestinal isolates from broilers. However, in isolates originating from meat, IncK plasmids were identified in all but one isolate (n = 101, 99%) with conjugative plasmids (Table 1). In addition to IncK and IncI1 plasmids, IncFII and IncFIB plasmids were identified in a few transconjugants (S2 Fig).

### *In silico* typing of WGS data

Isolates within the main PFGE cluster were identified as belonging to ST38 and serotype O7:H18. Five isolates with a highly similar PFGE pattern but grouping just outside the main PFGE cluster were also identified as belonging to ST38, serotype O7:H18. Isolates exhibiting nearly identical MLVA patterns (n = 5) belonged to ST1158 and serotype O17/77:H34. Among the ST38 strains, three isolates contained the *bla*_TEM-1_ gene in addition to *bla*_CMY-2._ No additional acquired resistance genes were identified in the remaining isolates subjected to WGS. The WGS data further showed that 14 isolates carried the virulence factors *iss* (increased serum survival) and *iha* (adhesion siderophore). Eleven isolates carried *gad* (glutamate decarboxylase), and nine isolates carried *iroN* (enterobactin siderophore) and *cma* (colicin M). Also, a few isolates carried *celb* (endonuclease colicin) and *astA* (heat-stable enterotoxin) [37, 38]. The SNP analysis (S5 Fig) showed that isolates within the main cluster, including both intestinal and meat isolates had a limited number of SNP differences (2–14 SNPs). Between isolates in the main cluster and isolates with highly similar PFGE banding patterns outside the main cluster (ST38), 138–150 SNP differences were observed. Isolates that were non-typeable by PFGE but displayed highly similar MLVA profiles (ST1158), differed by 8–63 SNPs. More than 10352 SNP differences were observed between isolates in the two main clusters defined by PFGE and MLVA (ST38 and ST1158) (S1 Table).

### Plasmid characterization

**Fig 1:**
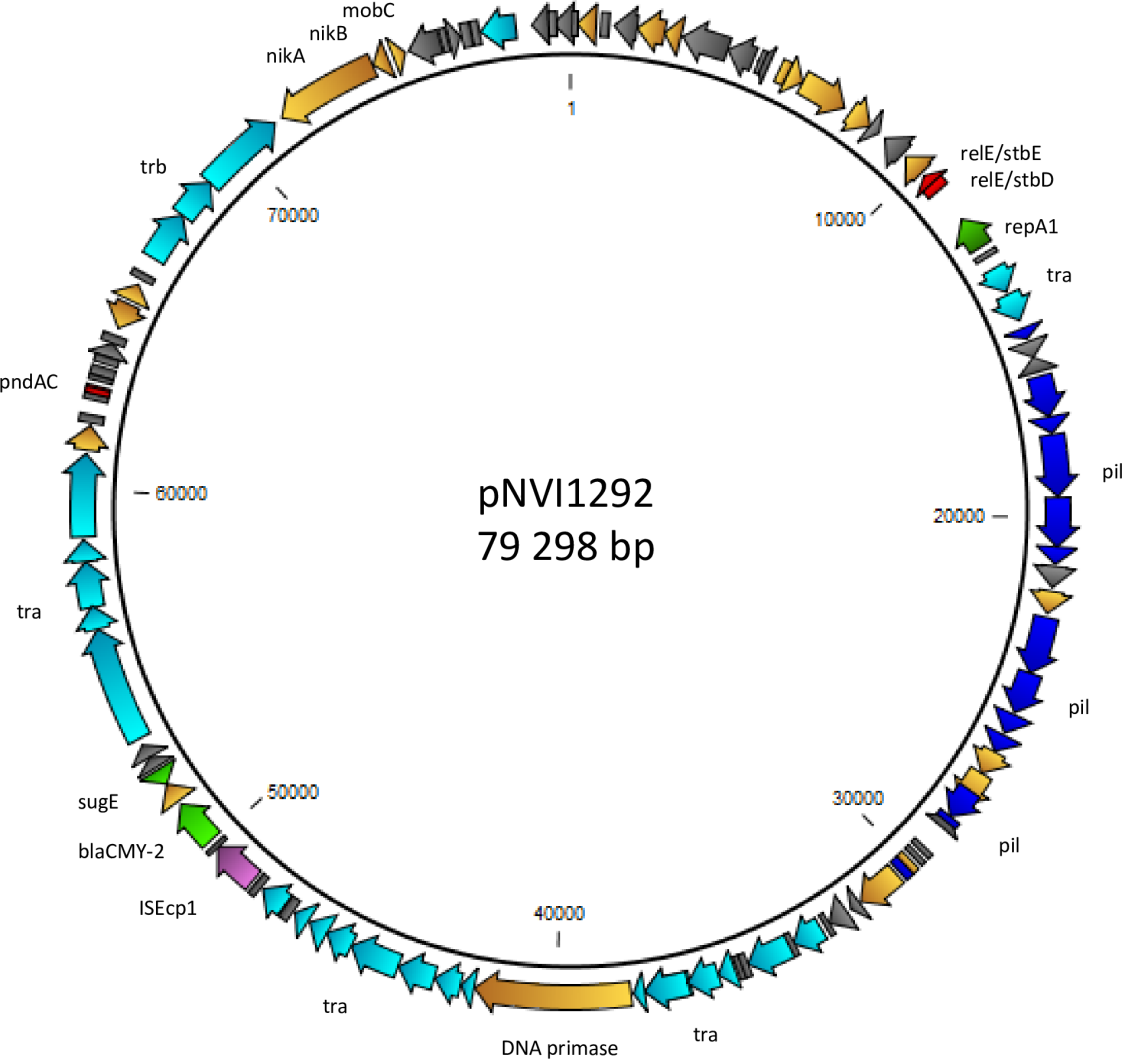
Structure of the *bla*_CMY-2_ containing plasmid pNVI1292 isolated from 1292DH5α. Light blue colour indicate transfer associated genes, blue colour indicate pilus associated genes, light green colour indicate resistance genes, red colour indicate plasmid stability systems, purple colour indicate insertion sequence, dark green colour indicate replication associated genes, orange colour indicate other proteins, and grey colour indicate hypothetical proteins.

**Fig 2:**
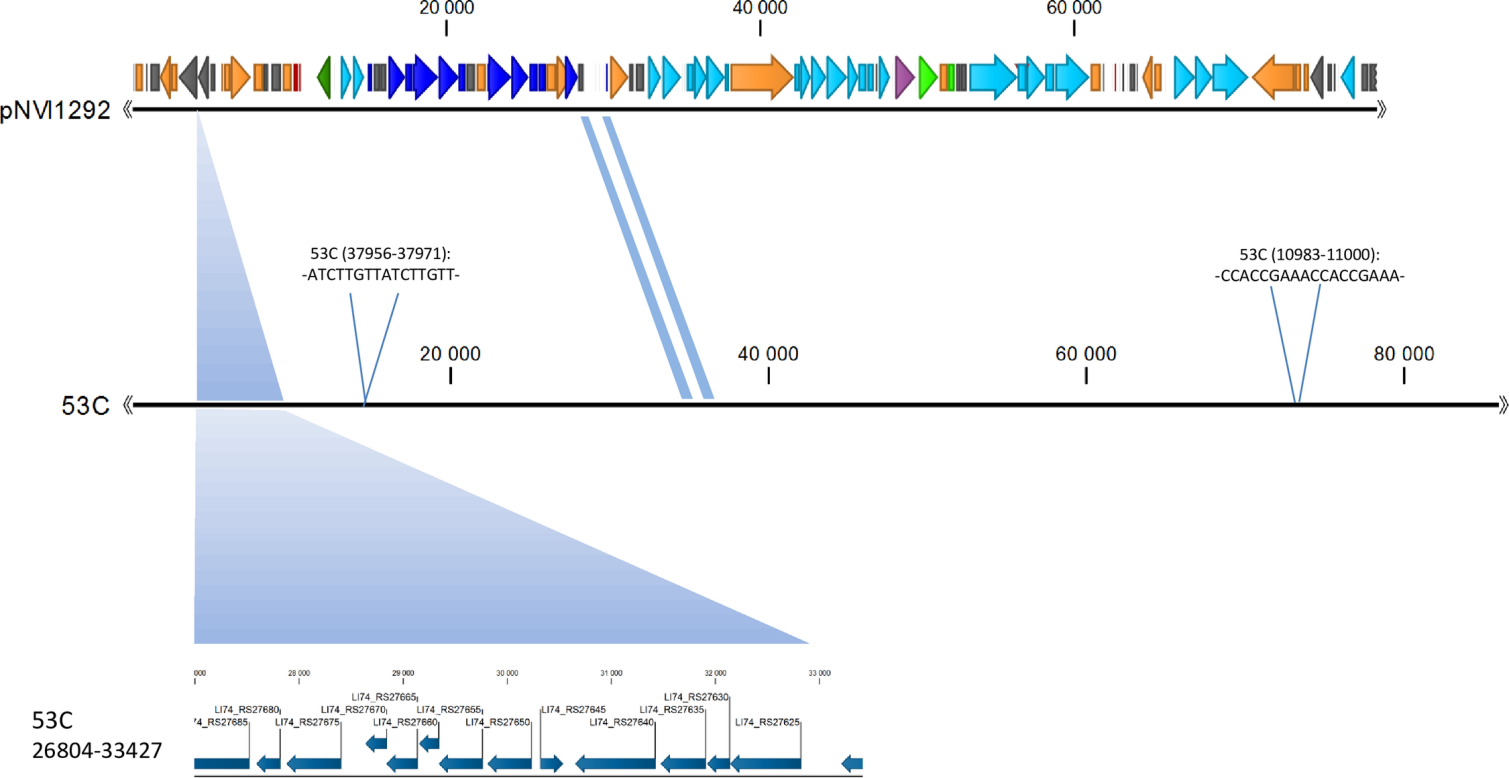
Alignment of plasmid pNVI1292 from 1292DH5α and *Escherichia coli* 53C. A 6.6 kB sequence present in the plasmid from *Escherichia coli* 53C but absent in the pNVI1292 plasmid is shown in the bottom of the figure. Differences in the nucleotide sequence between the two plasmids are marked with blue shadings.

The nucleotide sequence of an IncK plasmid harboured by an ST38 isolate grouping within the main PFGE cluster (1292DH5α), designated pNVI1292, was determined and characterized. The plasmid was 79.3 kB in size, and the majority of the genes encoded were associated with conjugal transfer or transcription. The *bla*_CMY-2_ gene was flanked by IS*Ecp1* downstream and *blc* and *sugE* upstream. This genetic organization has previously been described in other plasmids from *E*. *coli* and *Salmonella enterica* [39]. The insertion sequence IS*Ecp1* is known to function both as a transposase [40], and as a strong promotor for expression of genes located downstream [41]. The *blc* gene encodes a membrane lipoprotein [42], while *sugE* encodes an efflux pump mediating resistance to quaternary ammonium compounds when overexpressed [43]. In addition, two plasmid stability systems, namely *relBE/stbDE* and *pndAC* were present on the plasmid sequence (Fig 1). The plasmid sequence showed close homology to *Escherichia coli* 53C plasmid unnamed 3 (Accession number NZ_JXMX01000007.1) isolated from retail chicken meat in the Netherlands in 2010 [22, 25]. However, the pNVI1292 plasmid lacked a 6.6 kb sequence compared to the plasmid from the Netherlands (Fig 2). This 6.6 kb sequence was not found in any of the isolates subjected to WGS in this study.

The nucleotide sequence of the pNVI1292 plasmid from 1292DH5α has been submitted to GenBank (KU312044).

Analysis of the plasmid sequences from all isolates subjected to WGS (ST38 and ST1158) by the use of CSI Phylogeny showed that a 75.6 kb part of the nucleotide sequence from the pNVI1292 plasmid from 1292DH5α was present in all the isolates (S2 Table). This indicates that plasmids within ST38 and ST1158 share a high degree of similarity.

### Discussion

Our genotyping studies showed that cephalosporin resistant *E*. *coli* isolated from the intestinal flora of chickens in Norway is more diverse than those isolated from retail chicken meat. Two large clusters of genetically related isolates from meat were identified, belonging to ST38 and ST1158, respectively. In other studies, a more diverse genetic composition of cephalosporin-resistant *E*. *coli* has been identified in chicken meat samples [21, 23, 44, 45]. To our best knowledge, there are no other studies demonstrating the dominance of particular cephalosporin resistant clones in chicken meat over a prolonged time period. Only one pAmpC gene, namely *bla*_CMY-2_ was detected in cephalosporin resistant *E*. *coli* isolated from chicken and chicken meat in Norway [10]. Worldwide, *bla*_CMY-2_ is associated with pAmpC- producing *E*. *coli* isolated from poultry [3]. In Europe, *bla*_CMY-2_ is frequently linked to IncA/C and IncI1 plasmids in both human and poultry isolates [7], however an association with IncK plasmids has recently been reported [22, 24, 44, 46–48]. An equal distribution between IncK and IncI1 plasmids carrying *bla*_CMY-2_ was seen among pAmpC-producing *E*. *coli* isolated from faecal samples. However, in isolates originating from meat samples, the majority were found to carry IncK plasmids. This may be explained partly by the abundance of *E*. *coli* ST38 and ST1158 carrying IncK plasmids in Norway. SNP analysis of isolates subjected to WGS showed few SNP differences between ST38 isolates with highly similar PFGE banding patterns. Furthermore, few SNP differences between isolates originating from broilers and retail meat, and isolates collected in different years was seen. It is a perception that *E*. *coli* isolates from food-borne outbreaks have been shown to differ by 10–20 SNPs [49].Until now, clonal dissemination of cephalosporin-resistant *E*. *coli* has mostly been described for human adapted *E*. *coli* variants [8, 9]. However, our results showing few SNP differences highlight that clonal dissemination of pAmpC-producing strains has occurred in the Norwegian broiler production. Most of the *bla*_CMY-2_ carrying plasmids were conjugative *in vitro*. This indicates a large potential for horizontal transfer of these resistance plasmids in the bacterial population. The fact that the genetic diversity was smaller among meat isolates compared to intestinal isolates, and that IncK plasmids are dominating, indicate that a selection step favouring these variants and plasmids may occur from live animals to retail meat. Possibly, these variants and/or plasmids harbour a selection advantage throughout the production pyramid.

Our genotyping results revealed that a considerable proportion of the meat isolates belonged to ST38. This ST has also been identified in *E*. *coli* carrying *bla*_CMY-2_ on an IncK plasmid isolated from broilers and retail chicken meat in the Netherlands [22, 50], broilers and parent animals in Denmark [48], and retail chicken meat originating from Sweden, Finland and Denmark [46]. Thus, *E*. *coli* ST38 seems to be a variant commonly associated with cephalosporin resistance in the European broiler production pyramid. *E*. *coli* ST38 is associated with both uropathogenic *E*. *coli* (UPEC) and entero-aggregative *E*. *coli* (EAEC) in humans, but these isolates often carry other cephalosporin resistance genes on plasmids different than those found in isolates from broilers and retail chicken meat [51–53]. However, recent findings in Norway revealed a few *E*. *coli* ST38 carrying *bla*_CMY-2_ on IncK plasmids in human UPEC isolates. These isolates and plasmids were highly similar to isolates and plasmids from retail chicken meat. The results indicate that chicken meat may be a possible source for human acquisition of resistance plasmids and pAmpC-producing *E*. *coli* [54]. However, further studies are required to determine the route of transmission and whether other possible sources exist for human acquisition of these bacteria. Among isolates from 2014 we identified ST1158 as an emerging ST. Isolates within this ST were non-typeable by PFGE, but the majority displayed highly similar MLVA profiles. This finding may indicate a shift of the clonality of the pAmpC-producing *E*. *coli* isolated from chicken meat. This may be due to introduction of new pAmpC-producing *E*. *coli* strains through import of hatching eggs. ST38 was still prevalent in meat isolates from 2014. *E*. *coli* ST1158 has also been reported in retail chicken meat from Switzerland, but carrying *bla*_CTX-M-1_ and not *bla*_CMY-2_ [45]. To our best knowledge, this is the first report of *E*. *coli* ST1158 carrying *bla*_CMY-2_ on an IncK plasmid.

The pNVI1292 plasmid characterized in this study was highly similar to a plasmid isolated from *E*. *coli* in Dutch chicken meat in 2010. It may therefore be argued that this plasmid is disseminated and successful in the European broiler production. The pNVI1292 plasmid was isolated in 2012, and highly similar plasmids were identified in *E*. *coli* isolated from chicken in 2014. This suggests that the IncK plasmid has been circulating in the European broiler production for several years. Furthermore, the WGS data supported the hypothesis that related IncK plasmids are widespread and successful in the Norwegian broiler production. Overall, the results from the plasmid characterization and genotyping experiments strengthen the theory that import of hatching eggs is the original source of pAmpC-producing *E*. *coli* in the Norwegian broiler production.

The presence of the plasmid stability systems *relBE/stbDE* and *pndAC* represents a selection advantage for the plasmids due to post-segregational killing of plasmid-free daughter cells. Also, the addiction modules ensure plasmid stability during replication by elimination of other compatible plasmids from the bacteria [55]. This may represent an explanation of how pAmpC-producing *E*. *coli* can be maintained in the Norwegian broiler production in spite of the absence of selection pressure from antimicrobial use.

Phylotyping revealed that the majority of the isolates from both broilers and retail chicken meat belonged to phylogroup D. Also, a small number of isolates were identified as belonging to phylogroup B2. A small study from Denmark also showed that phylogroup D dominated among *E*. *coli* isolated from the broiler production pyramid and retail meat [48]. Also, phylogroup D and *bla*_CMY-2_ has previously been shown to have a close association with *E*. *coli* isolated from poultry in Spain [56]. The phylogroups D and B2 are associated with human extraintestinal pathogenic *E*. *coli* (ExPEC) strains [57]. Also, ExPEC associated virulence factors were detected among isolates subjected to WGS, namely *iss*, *iha*, *iroN* and *astA* [58, 59]. Thus, there are indications that pAmpC-producing *E*. *coli* from broilers and retail chicken meat in Norway may have pathogenic potential.

In conclusion, both clonal expansion and horizontal transfer of *bla*_CMY-2_ carrying plasmids are likely to contribute to the dissemination of pAmpC-producing *E*. *coli* in the Norwegian broiler production. The presence of the plasmid stability systems *relBE/stbDE* and *pndAC* is likely to play a significant role in the broad dissemination and maintenance of IncK plasmids carrying *bla*_CMY-2_, as it represents a selection advantage for isolates carrying this plasmid. We also suggest that highly related *bla*_CMY-2_ carrying IncK plasmids are disseminated and successful in the European broiler production.

## Supporting Information

S1 Fig Pulsed-Field gel electrophoresis (PFGE) banding patterns for intestinal isolates. Dendrogram based on UPGMA cluster analysis of PFGE banding patterns of pAmpC-producing *E*. *coli* isolated from and faecal samples from healthy broiler chickens, 2011. The red line indicates 80% similarity of banding patterns, and the blue line indicates 97% similarity of banding patterns. Isolates subjected to whole genome sequencing are indicated by *. Isolates that were PFGE non-typeable are shown as a straight line in the bottom of the dendrogram.
(PDF)

S2 Fig Pulsed-Field gel electrophoresis (PFGE) banding patterns for meat isolates. Dendrogram based on UPGMA cluster analysis of PFGE banding patterns of pAmpC- producing *E*. *coli* isolated from retail chicken meat, 2012 and 2014. The red line indicates 80% similarity of banding patterns, and the blue line indicates 97% similarity of banding patterns. Isolates subjected to whole genome sequencing are indicated by *. Isolates that were PFGE non-typeable are shown as a straight line in the bottom of the dendrogram.
(PDF)

S3 Fig Dendrogram including Pulsed-Field gel electrophoresis (PFGE) banding patterns of all isolates included in the study. The red line denotes 80% similarity of banding patterns, while the blue line denotes 97% similarity of banding patterns. The green box highlights the main cluster including 69 isolates. Isolates that were PFGE non-typeable are shown as a straight line in the bottom of the dendrogram. Isolates subjected to whole genome sequencing are indicated by *.
(PDF)

S4 Fig Minimum-spanning-tree analysis of 20 meat isolates subjected to Multiple locus variable-number tandem-repeats analysis (MLVA). Green colour represents isolates belonging to phylogroup D, while blue colour represents isolates belonging to phylotype B1. The main cluster of highly related MLVA profiles, including 15 isolates, is indicated by the red circle.
(TIF)

S5 Fig Single nucleotide polymorphism (SNP) tree of isolates subjected to whole genome sequencing and NCBI *E*. *coli* reference genomes. Retail meat isolates are highlighted with yellow, isolates from broiler faeces are highlighted with green, while NCBI *E*. *coli* reference genomes are highlighted with blue. Isolate 2012-01-1292 was used as reference. Isolates 2012-01-1292ctrl and 2011-01-2112-2ctrl were included as control of sequence reproducibility.
(PDF)

S1 Table Number of single nucleotide polymorphisms (SNPs) between pairs of isolates subjected to whole genome sequencing. Isolate 2012-01-1292 was used as reference. Isolates 2012-01-1292ctrl and 2011-01-2112-2ctrl were included as control of sequence reproducibility.
(XLSX)

S2 Table Percentage similarity between IncK plasmids subjected to whole genome sequencing. The assembled plasmid sequence from pNVI1292 was used as reference (79.3 kb). Percentage of reference genome covered by all isolates was 95.3% (75.6 kb). Number of single nucleotide polymorphisms (SNPs) between pairs of plasmids is shown.
(XLSX)
