# Supplementary material for: Plasmid and Host Strain Characteristics of Escherichia coli Resistant to Extended-Spectrum Cephalosporins in the Norwegian Broiler Production
Source: PLoS One. 2016 Apr 25;11(4):e0154019. doi: 10.1371/journal.pone.0154019 (PMC4844124; doi:10.1371/journal.pone.0154019)
Supplement: S2 Fig — Dendrogram based on UPGMA cluster analysis of PFGE banding patterns of pAmpC- producing E. coli isolated from retail chicken meat, 2012 and 2014. The red line indicates 80% similarity of banding patterns, and the blue line indicates 97% similarity of banding patterns. Isolates subjected to whole genome sequencing are indicated by *. Isolates that were PFGE non-typeable are shown as a straight line in the bottom of the dendrogram. (PDF) [file pone.0154019.s002.pdf]

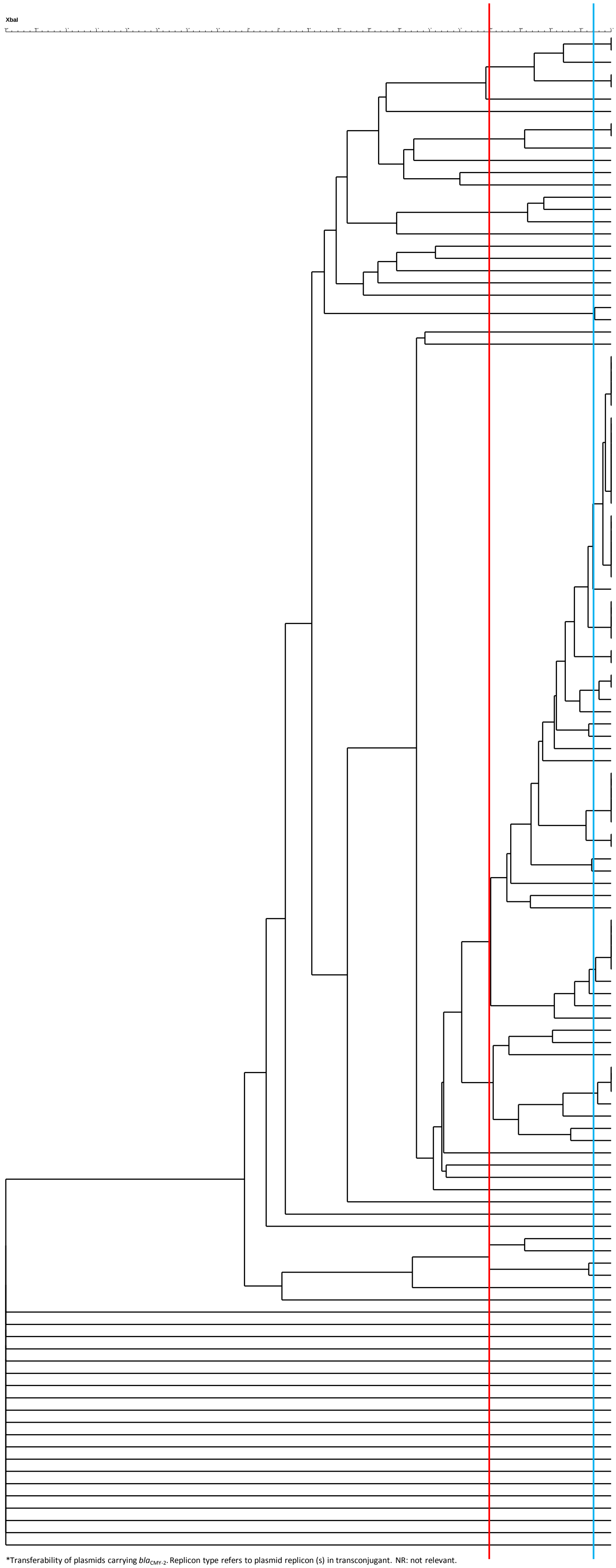

ID	Source	Year	Phylogroup	Conjugative plasmid*	Replicontype*
2014-01-2449	Meat	2014	D	-	NR
2014-01-2452	Meat	2014	D	-	NR
2014-01-5687	Meat	2014	D	-	NR
2014-01-5983	Meat	2014	D	-	NR
2014-01-6137	Meat	2014	D	-	NR
2014-01-1676	Meat	2014	D	-	NR
2012-01-2798	Meat	2012	A	+	I1
2014-01-7010	Meat	2014	B2	+	K
2014-01-7037	Meat	2014	B2	+	K
2014-01-6321	Meat	2014	B2	+	K
2014-01-7011	Meat	2014	D	-	NR
2014-01-4267	Meat	2014	A	+	K
2014-01-7150	Meat	2014	B2	+	K
2014-01-5104	Meat	2014	D	+	K
2014-01-5689	Meat	2014	D	+	K
2014-01-1672	Meat	2014	D	+	K
2012-01-5334	Meat	2012	A	+	K
2014-01-1336	Meat	2014	A	+	K, I1
2014-01-7427	Meat	2014	B2	+	K
2014-01-1673	Meat	2014	B1	-	NR
2014-01-3681	Meat	2014	B1	-	NR
2014-01-4991	Meat	2014	D	+	K
2012-01-771	Meat	2012	D	+	K
2012-01-960	Meat	2012	D	+	K
2012-01-7896	Meat	2012	D	+	K
2014-01-2454	Meat	2014	D	-	NR
2012-01-1137	Meat	2012	D	+	K, FIB, FII
2012-01-5335	Meat	2012	D	+	K
2012-01-1292 *	Meat	2012	D	+	K
2012-01-1459	Meat	2012	D	+	K
2012-01-1988	Meat	2012	D	+	K
2012-01-772	Meat	2012	D	+	K
2012-01-1138	Meat	2012	D	+	K
2012-01-1293	Meat	2012	D	-	NR
2012-01-1294	Meat	2012	D	+	K
2012-01-1657	Meat	2012	D	+	K
2012-01-2350	Meat	2012	D	+	K
2012-01-3380	Meat	2012	D	+	K
2012-01-5336	Meat	2012	D	+	K
2012-01-3702	Meat	2012	D	+	K
2012-01-4274	Meat	2012	D	+	K
2012-01-3703	Meat	2012	D	+	K
2012-01-4101	Meat	2012	D	+	K
2012-01-4272	Meat	2012	D	+	K
2012-01-4273	Meat	2012	D	+	K
2012-01-5581	Meat	2012	D	+	K
2012-01-2800	Meat	2012	D	+	K
2012-01-6775	Meat	2012	D	+	K
2012-01-3701	Meat	2012	D	+	K
2012-01-6612	Meat	2012	D	-	NR
2012-01-3584	Meat	2012	D	+	K
2012-01-3585	Meat	2012	D	-	NR
2012-01-1134	Meat	2012	D	+	K, FIB, FII
2012-01-3686	Meat	2012	D	+	K
2012-01-2599	Meat	2012	D	+	K
2012-01-1550	Meat	2012	D	+	K
2012-01-706	Meat	2012	D	+	K
2012-01-1295	Meat	2012	D	+	K, FIB, FII
2012-01-708	Meat	2012	D	+	K
2014-01-6135	Meat	2014	D	-	NR
2012-01-6001 *	Meat	2012	D	+	K
2012-01-8642	Meat	2012	D	+	K
2012-01-7316	Meat	2012	D	+	K
2012-01-7898	Meat	2012	D	+	K
2012-01-7900	Meat	2012	D	+	K
2012-01-5998	Meat	2012	D	+	K
2012-01-7901	Meat	2012	D	+	K
2012-01-6154	Meat	2012	D	+	K
2014-01-5749	Meat	2014	D	-	NR
2012-01-707	Meat	2012	D	+	K
2014-01-7292	Meat	2014	D	+	K
2014-01-7296	Meat	2014	D	+	K
2012-01-1552	Meat	2012	D	-	NR
2012-01-1553 *	Meat	2012	D	+	K
2012-01-1849 *	Meat	2012	D	+	K
2012-01-2348	Meat	2012	D	+	K
2012-01-5332	Meat	2012	D	+	K
2012-01-5507	Meat	2012	D	+	K
2012-01-3184	Meat	2012	D	+	K
2012-01-5333	Meat	2012	D	+	K
2012-01-6774	Meat	2012	D	+	K
2012-01-6613	Meat	2012	D	+	K
2014-01-7008	Meat	2014	D	+	K
2014-01-3384	Meat	2014	D	+	K
2014-01-14	Meat	2014	D	+	K
2014-01-1040 *	Meat	2014	D	+	K
2014-01-1044 *	Meat	2014	D	+	K
2014-01-1675 *	Meat	2014	D	+	K, FIB, FII
2014-01-4662	Meat	2014	D	-	NR
2014-01-1076	Meat	2014	D	+	K
2014-01-5572	Meat	2014	D	-	NR
2014-01-5771	Meat	2014	D	-	NR
2012-01-4096	Meat	2012	D	+	K
2012-01-6908	Meat	2012	D	+	K
2012-01-646	Meat	2012	D	+	K
2014-01-7295	Meat	2014	D	+	K
2012-01-3586	Meat	2012	B2	+	K
2012-01-3762	Meat	2012	D	+	K
2012-01-5997	Meat	2012	A	+	K
2014-01-5656	Meat	2014	A	+	K
2012-01-1658	Meat	2012	A	+	K
2012-01-1659	Meat	2012	A	+	K
2014-01-7149	Meat	2014	A	+	K
2014-01-7282	Meat	2014	D	+	K
2012-01-6611	Meat	2012	D	+	K
2014-01-13 *	Meat	2014	D	+	K
2014-01-1042 *	Meat	2014	D	+	K
2014-01-1043 *	Meat	2014	D	+	K
2014-01-1756	Meat	2014	D	-	NR
2014-01-3128	Meat	2014	D	+	K
2014-01-3678	Meat	2014	D	+	K
2014-01-3680 *	Meat	2014	D	+	K
2014-01-3682	Meat	2014	D	-	NR
2014-01-3683	Meat	2014	D	-	NR
2014-01-4268	Meat	2014	D	+	K
2014-01-5100	Meat	2014	A	+	K
2014-01-5105 *	Meat	2014	D	+	K, FII
2014-01-5106	Meat	2014	D	+	K
2014-01-5686	Meat	2014	D	+	K
2014-01-5750	Meat	2014	A	+	K
2014-01-5772	Meat	2014	D	+	K
2014-01-5982	Meat	2014	D	+	K
2014-01-6247	Meat	2014	A	+	K
2014-01-7475	Meat	2014	D	+	K

*Transferability of plasmids carrying *bla*$_{CMY-2}$. Replicon type refers to plasmid replicon (s) in transconjugant. NR: not relevant.
