# Supplementary figures and images for: Plasmid and Host Strain Characteristics of Escherichia coli Resistant to Extended-Spectrum Cephalosporins in the Norwegian Broiler Production
Source: PLoS One. 2016 Apr 25;11(4):e0154019. doi: 10.1371/journal.pone.0154019 (PMC4844124; doi:10.1371/journal.pone.0154019)

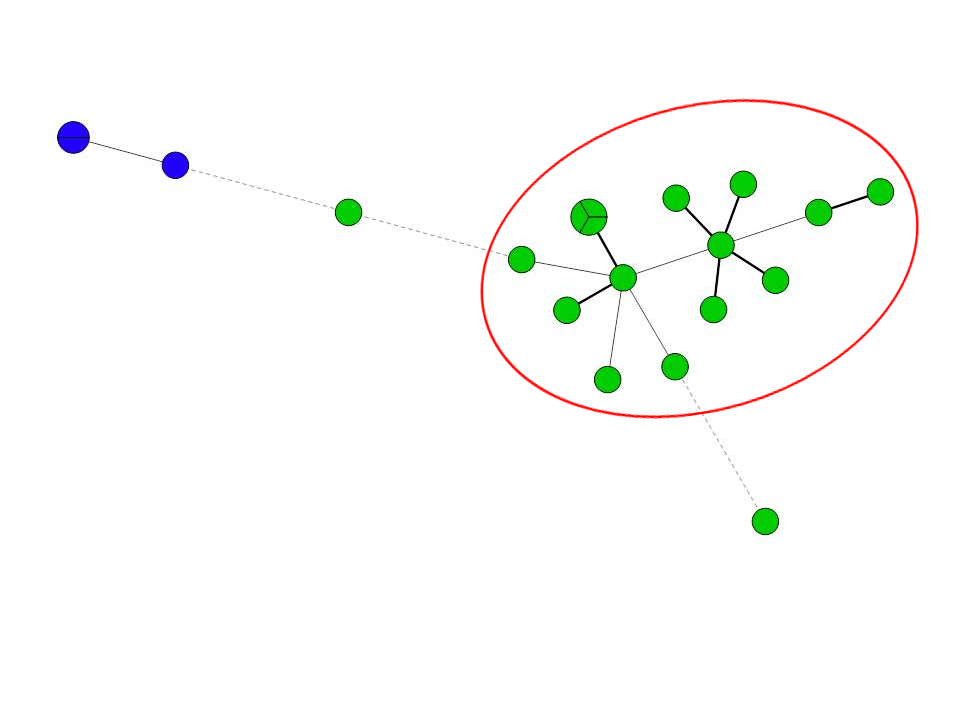

Supplement: S4 Fig — Green colour represents isolates belonging to phylogroup D, while blue colour represents isolates belonging to phylotype B1. The main cluster of highly related MLVA profiles, including 15 isolates, is indicated by the red circle. (TIF) [file pone.0154019.s004.tif]
