# Supplementary material for: Plasmid and Host Strain Characteristics of Escherichia coli Resistant to Extended-Spectrum Cephalosporins in the Norwegian Broiler Production
Source: PLoS One. 2016 Apr 25;11(4):e0154019. doi: 10.1371/journal.pone.0154019 (PMC4844124; doi:10.1371/journal.pone.0154019)
Supplement: S5 Fig — Retail meat isolates are highlighted with yellow, isolates from broiler faeces are highlighted with green, while NCBI E. coli reference genomes are highlighted with blue. Isolate 2012-01-1292 was used as reference. Isolates 2012-01-1292ctrl and 2011-01-2112-2ctrl were included as control of sequence reproducibility. (PDF) [file pone.0154019.s005.pdf]

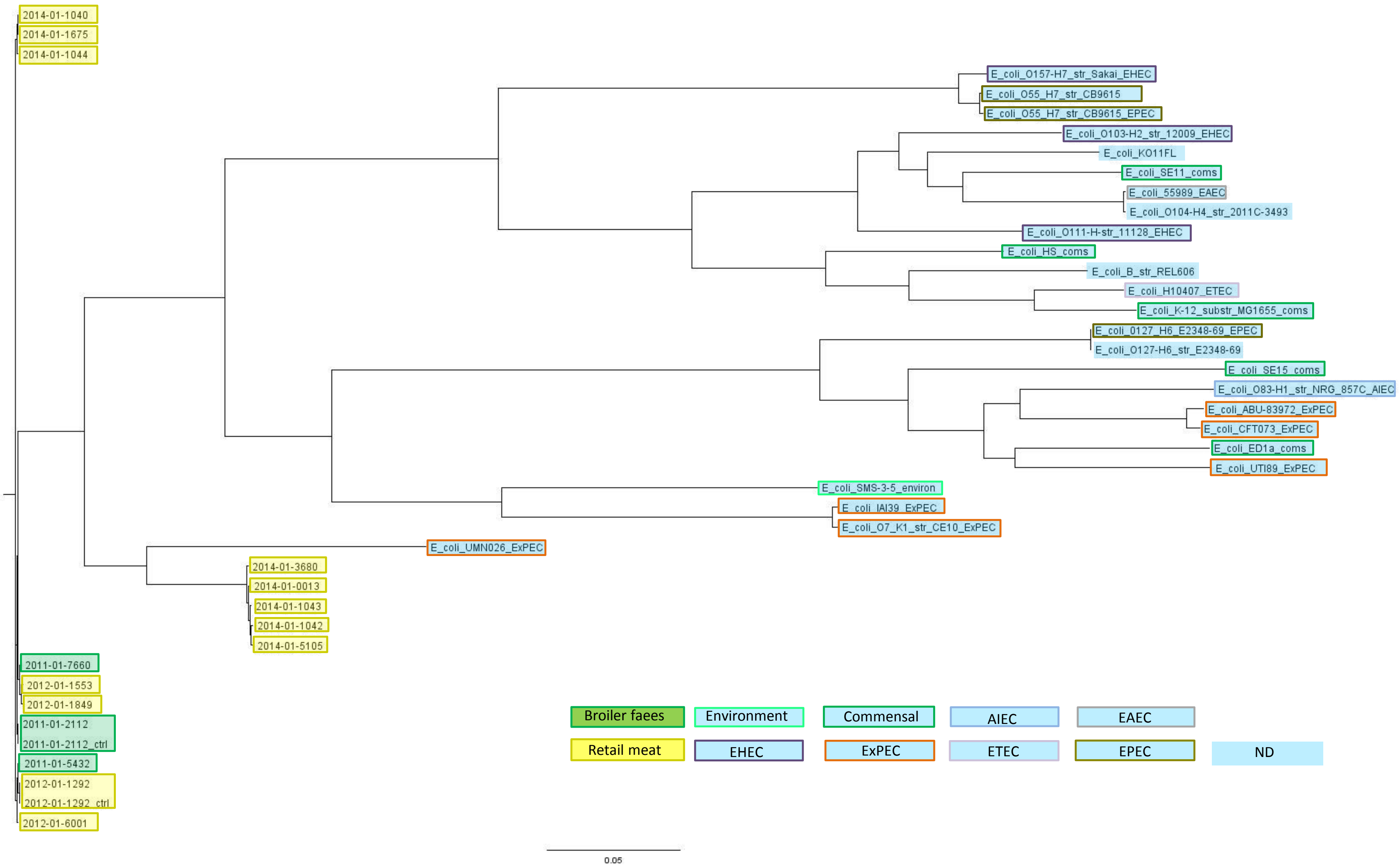

2014-01-1040
2014-01-1675
2014-01-1044
E_coli_O157-H7_str_Sakai_EHEC
E_coli_O55_H7_str_CB9615
E_coli_O55_H7_str_CB9615_EPEC
E_coli_O103-H2_str_12009_EHEC
E_coli_KO11FL
E_coli_SE11_coms
E_coli_55989_EAEC
E_coli_O104-H4_str_2011C-3493
E_coli_O111-H-str_11128_EHEC
E_coli_HS_coms
E_coli_B_str_REL606
E_coli_H10407_ETEC
E_coli_K-12_substr_MG1655_coms
E_coli_O127_H6_E2348-69_EPEC
E_coli_O127-H6_str_E2348-69
E_coli_SE15_coms
E_coli_O83-H1_str_NRG_857C_AIEC
E_coli_ABU-83972_ExPEC
E_coli_CFT073_ExPEC
E_coli_ED1a_coms
E_coli_UTI89_ExPEC
E_coli_SMS-3-5_environ
E_coli_IAI39_ExPEC
E_coli_O7_K1_str_CE10_ExPEC
E_coli_UMN026_ExPEC
2014-01-3680
2014-01-0013
2014-01-1043
2014-01-1042
2014-01-5105
2011-01-7660
2012-01-1553
2012-01-1849
2011-01-2112
2011-01-2112_ctrl
2011-01-5432
2012-01-1292
2012-01-1292_ctrl
2012-01-6001
Broiler faees
Environment
Commensal
AIEC
EAEC
Retail meat
EHEC
ExPEC
ETEC
EPEC
ND
0.05
