# Supplementary material for: Plasmid and Host Strain Characteristics of Escherichia coli Resistant to Extended-Spectrum Cephalosporins in the Norwegian Broiler Production
Source: PLoS One. 2016 Apr 25;11(4):e0154019. doi: 10.1371/journal.pone.0154019 (PMC4844124; doi:10.1371/journal.pone.0154019)
Supplement: S1 Fig — Dendrogram based on UPGMA cluster analysis of PFGE banding patterns of pAmpC-producing E. coli isolated from and faecal samples from healthy broiler chickens, 2011. The red line indicates 80% similarity of banding patterns, and the blue line indicates 97% similarity of banding patterns. Isolates subjected to whole genome sequencing are indicated by *. Isolates that were PFGE non-typeable are shown as a straight line in the bottom of the dendrogram. (PDF) [file pone.0154019.s001.pdf]

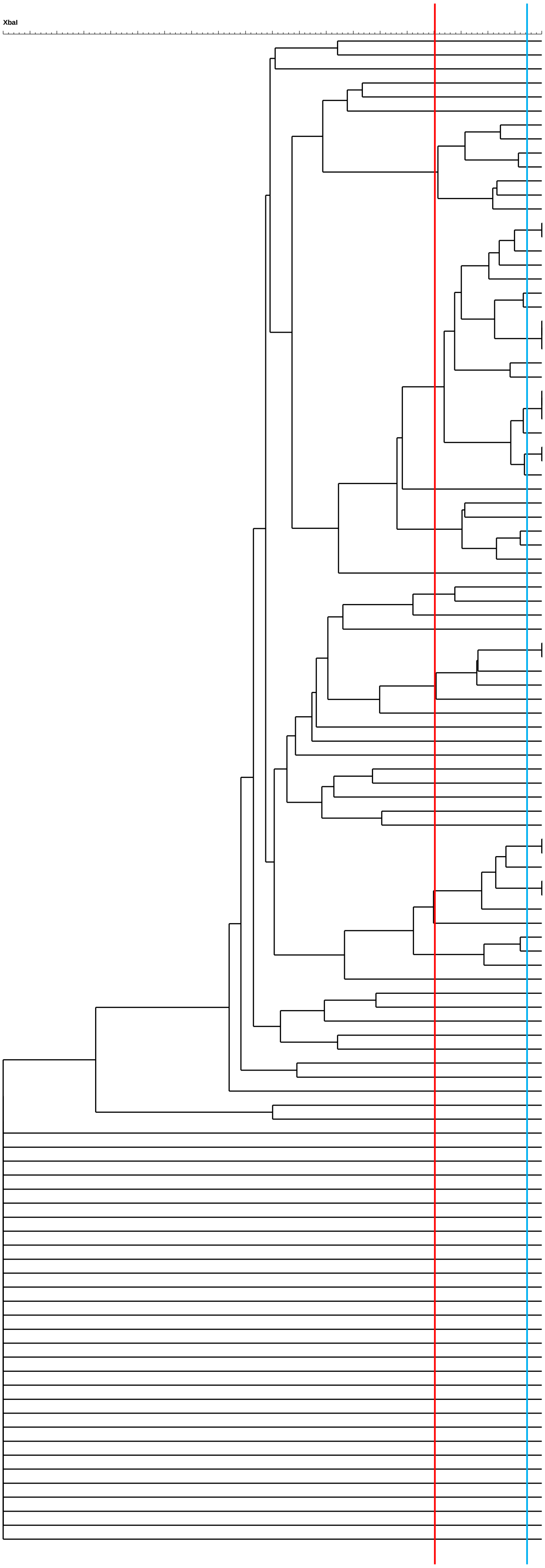

ID	Source	Year	Phylogroup	Conjugative plasmid*	Replicontype*
2011-01-323-4	Faeces	2011	D	+	I1
2011-01-7660-2	Faeces	2011	D	+	K
2011-01-3018-3	Faeces	2011	D	+	K
2011-01-3460-1	Faeces	2011	D	+	K
2011-01-8908-6	Faeces	2011	D	+	K
2011-01-3460-3	Faeces	2011	B1	+	K, FIB, FII
2011-01-3018-6	Faeces	2011	B1	+	I1
2011-01-5251-1	Faeces	2011	B1	+	I1, FII
2011-01-5432-6	Faeces	2011	B1	+	I1
2011-01-5610-1	Faeces	2011	B1	+	I1
2011-01-6462-5	Faeces	2011	B1	+	I1
2011-01-7660-5	Faeces	2011	B1	+	I1
2011-01-5252-1	Faeces	2011	B1	+	I1
2011-01-7660-1 *	Faeces	2011	D	+	K
2011-01-8304-4	Faeces	2011	D	+	K
2011-01-5252-4	Faeces	2011	D	+	K
2011-01-8709-3	Faeces	2011	D	+	K
2011-01-6227-2	Faeces	2011	D	+	K
2011-01-3460-4	Faeces	2011	D	+	K
2011-01-8174-1	Faeces	2011	D	+	K
2011-01-3717-6	Faeces	2011	D	+	K
2011-01-3991-2	Faeces	2011	D	+	K
2011-01-3991-6	Faeces	2011	D	+	K
2011-01-7660-6	Faeces	2011	D	+	K
2011-01-8908-2	Faeces	2011	D	+	K
2011-01-5432-1	Faeces	2011	D	+	K
2011-01-5432-5	Faeces	2011	D	+	K
2011-01-6462-1	Faeces	2011	D	+	K
2011-01-2112-2 *	Faeces	2011	D	+	K
2011-01-5432-3 *	Faeces	2011	D	+	K
2011-01-5815-4	Faeces	2011	D	+	K
2011-01-7378-5	Faeces	2011	D	+	K
2011-01-7660-3	Faeces	2011	D	+	K
2011-01-101-4	Faeces	2011	D	+	K
2011-01-4277-3	Faeces	2011	D	+	K
2011-01-4277-2	Faeces	2011	D	+	K, FIB, FII
2011-01-5250-6	Faeces	2011	D	+	K
2011-01-5250-7	Faeces	2011	D	+	K
2011-01-8304-5	Faeces	2011	D	+	I1
2011-01-5815-5	Faeces	2011	B2	+	I1
2011-01-6928-5	Faeces	2011	B1	-	NR
2011-01-7940-6	Faeces	2011	B2	+	I1
2011-01-6927-1	Faeces	2011	D	+	I1
2011-01-1670-3	Faeces	2011	B1	-	NR
2011-01-1670-5	Faeces	2011	B1	+	K
2011-01-1544-4	Faeces	2011	B1	-	NR
2011-01-1544-6	Faeces	2011	B1	+	I1
2011-01-6928-3	Faeces	2011	B1	-	NR
2011-01-6928-6	Faeces	2011	B2	+	I1
2011-01-1960-3	Faeces	2011	A	+	K
2011-01-323-3	Faeces	2011	B2	+	K
2011-01-6462-6	Faeces	2011	D	+	I1
2011-01-323-5	Faeces	2011	B2	+	K, I1
2011-01-7940-2	Faeces	2011	D	+	K, I1
2011-01-8304-1	Faeces	2011	D	+	K, I1
2011-01-1670-4	Faeces	2011	D	+	K
2011-01-3646-6	Faeces	2011	A	+	I1
2011-01-5610-4	Faeces	2011	B1	+	I1
2011-01-6928-1	Faeces	2011	B1	+	I1
2011-01-3646-4	Faeces	2011	B1	+	I1
2011-01-5254-5	Faeces	2011	B1	+	I1
2011-01-5815-2	Faeces	2011	B1	+	I1
2011-01-5610-3	Faeces	2011	B1	+	I1
2011-01-3646-1	Faeces	2011	B1	+	I1
2011-01-1960-6	Faeces	2011	B1	+	I1
2011-01-3717-1	Faeces	2011	B1	+	I1
2011-01-3717-3	Faeces	2011	B1	+	I1
2011-01-7464-6	Faeces	2011	B2	+	I1
2011-01-5251-4	Faeces	2011	D	+	I1
2011-01-8709-5	Faeces	2011	A	-	NR
2011-01-4277-4	Faeces	2011	B1	+	I1
2011-01-5251-5	Faeces	2011	A	+	I1
2011-01-6927-5	Faeces	2011	D	+	I1
2011-01-4182-2	Faeces	2011	A	+	I1
2011-01-7464-4	Faeces	2011	B1	+	I1
2011-01-5253-5	Faeces	2011	D	+	K
2011-01-7464-3	Faeces	2011	D	+	I1
2011-01-7660-4	Faeces	2011	D	+	I1
2011-01-101-5	Faeces	2011	D	+	I1
2011-01-1960-4	Faeces	2011	D	+	I1
2011-01-2112-3	Faeces	2011	A	+	K
2011-01-2112-5	Faeces	2011	D	+	I1
2011-01-2508-3	Faeces	2011	D	+	I1
2011-01-2508-6	Faeces	2011	D	+	I1
2011-01-3339-2	Faeces	2011	D	+	I1
2011-01-3339-5	Faeces	2011	D	+	I1
2011-01-3646-3	Faeces	2011	D	+	I1
2011-01-3717-2	Faeces	2011	D	+	I1
2011-01-3717-4	Faeces	2011	D	+	I1
2011-01-3717-5	Faeces	2011	D	+	I1
2011-01-4182-3	Faeces	2011	D	-	NR
2011-01-4182-4	Faeces	2011	A	-	NR
2011-01-4277-1	Faeces	2011	B1	-	NR
2011-01-5250-4	Faeces	2011	B2	+	K, I1
2011-01-5251-6	Faeces	2011	B1	+	K
2011-01-5254-1	Faeces	2011	D	+	I1
2011-01-5254-6	Faeces	2011	D	+	I1
2011-01-5815-1	Faeces	2011	A	+	K
2011-01-6227-6	Faeces	2011	D	+	K, I1
2011-01-6462-4	Faeces	2011	A	+	K, I1
2011-01-7378-4	Faeces	2011	D	+	I1
2011-01-7464-1	Faeces	2011	D	+	I1, I2
2011-01-7464-5	Faeces	2011	D	+	K
2011-01-7940-1	Faeces	2011	B1	+	K
2011-01-8174-2	Faeces	2011	D	+	I1
2011-01-8174-5	Faeces	2011	B1	+	K
2011-01-8908-5	Faeces	2011	D	+	I1
2011-01-9386-2	Faeces	2011	B1	+	K

*Transferability of plasmids carrying bla_{CMY-2}. Replicon type refers to plasmid replicon (s) in transconjugant. NR: not relevant.
