# Supplementary material for: Plasmid and Host Strain Characteristics of Escherichia coli Resistant to Extended-Spectrum Cephalosporins in the Norwegian Broiler Production
Source: PLoS One. 2016 Apr 25;11(4):e0154019. doi: 10.1371/journal.pone.0154019 (PMC4844124; doi:10.1371/journal.pone.0154019)
Supplement: S3 Fig — The red line denotes 80% similarity of banding patterns, while the blue line denotes 97% similarity of banding patterns. The green box highlights the main cluster including 69 isolates. Isolates that were PFGE non-typeable are shown as a straight line in the bottom of the dendrogram. Isolates subjected to whole genome sequencing are indicated by *. (PDF) [file pone.0154019.s003.pdf]

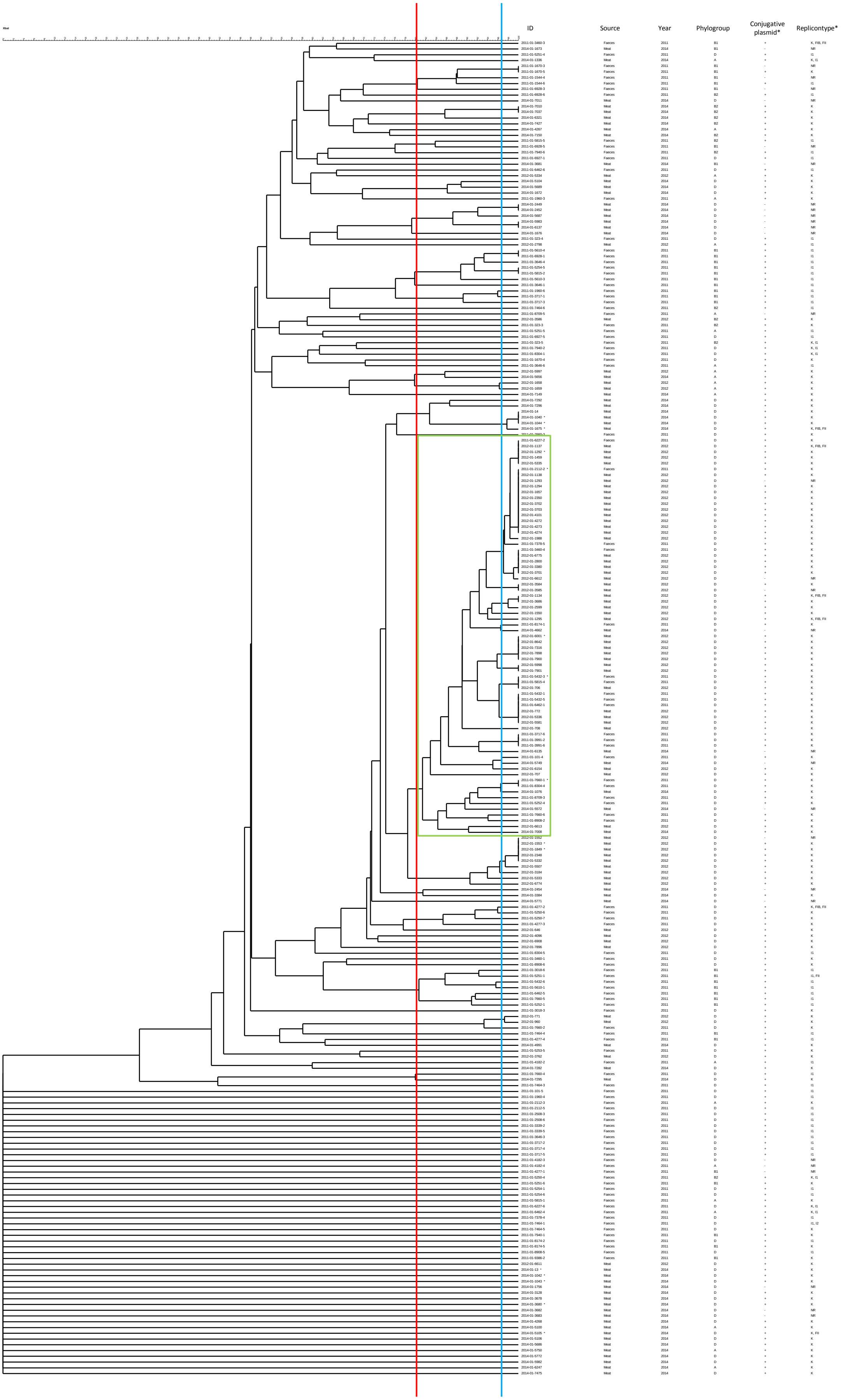

*Transferability of plasmids carrying bla_{CMY-2}. Replicon type refers to plasmid replicon (s) in transconjugant. NR: not relevant.
